# Past, Present, and Future of Viral Hepatitis in Bangladesh

**DOI:** 10.5005/jp-journals-10018-1164

**Published:** 2016-07-09

**Authors:** Mamun-Al-Mahtab

**Affiliations:** Department of Hepatology, Bangabandhu Sheikh Mujib Medical University, Dhaka, Bangladesh

**Keywords:** Bangladesh, Hepatitis B virus, Hepatitis C virus, Hepatitis E virus.

## Abstract

Viral hepatitis poses huge burden to the health care delivery system as well as to the economy of Bangladesh. Hepatitis E virus is the leading cause of acute hepatitis in this country, however with the improvement of economic status and sanitation this seems to be on the decline. Hepatitis B virus remains the leading cause in all forms of chronic liver diseases in this country. This virus has been extensively studied in Bangladesh, including from epidemiology to pathogenosis. Clinical trials with innovative therapy conducted in Bangladesh have shown promise. Hepatitis C virus follows hepatitis B virus as the second commonest cause of chronic liver diseases here. However patients in Bangladesh have started benefitting from the local generic versions of the newly introduced direct acting anti-virals.

**How to cite this article:**

Al-Mahtab M. Past, Present, and Future of Viral Hepatitis in Bangladesh. Euroasian J Hepato-Gastroenterol 2016;6(1):43-44.

## INRODUCTION

Bangladesh is a South Asian country with India on its 3 sides and the Bay of Bengal on the fourth side and a small land boundary with Myanmar shared with India on 1 side. The land area of Bangladesh is approximately 1,55,000 km^2^ and its population is around 160 million. Hepatitis viruses constitute the mainstay of liver diseases in Bangladesh with hepatitis B, C, and E contributing the most.

## HEPATITIS B VIRUS

Bangladesh is in the intermediate prevalence zone of hepatitis B virus (HBV) with an estimated prevalence of 5.4% in the general population.^1^ The main risk factors for the transmission of HBV in Bangladesh have been identified as treatment from quacks, shaving and haircut in barber shops, body piercing, as well as vaccination against small pox, cholera, dental procedure, intravenous infusion, etc.^1^ Mass vaccination against HBV was introduced in the expanded program of immunization (EPI) schedule for newborns in Bangladesh in 2003 with coverage of more than 97%. This has yielded positive outcome as the prevalence had declined from 8% in 1984 to 5.4% in 2007 in this country.^1^

However, HBV is still the leading cause of acute and chronic liver diseases (CLD) in Bangladesh. It is responsible for approximately 30% cases of acute hepatitis,^2^ 75% cases of chronic hepatitis,^3^ 60% cases of liver cirrhosis, and 65%^4^ cases of hepatocellular carcinoma (HCC)^5^ in Bangladesh.

The treatment of HBV is cheaper in Bangladesh compared to many other countries of the region, given the fact that we enjoy local, generics for anti-HBV drugs, i.e., tenofovir, entecavir, adefovir, lamivudine, and pegylated interferon (Peg IFN), with the exception of only telbivudine. However, as we more often see HBeAg-negative HBV infection in Bangladesh with the principal HBV genotype (GT) being GT C, the management of HBV remains a challenge for us.

Groundbreaking research is being conducted in Bangladesh for the development of new drugs against HBV, which include phase I/II and subsequent phase III studies with a therapeutic vaccine called NASVAC, comprising HBsAg and HBcAg and another nucleic acid polymer called Rep 09C that inhibits HBsAg release from infected hepatocytes. NASVAC, in particular, needs special mention, as it is the only immune therapy not only for HBV but for any chronic infection that has successfully gone through a phase III clinical trial; this has established its superiority over Peg IFN,^6^ prompting much enthusiasm globally as well as multicenter European and Asia Pacific clinical trials. Both drugs are expected in the market before the turn of the current decade.

## HEPATITIS C VIRUS

There is limited data about the prevalence of hepatitis C virus (HCV) in Bangladesh. Most of the studies are also carried out in limited population. Published literature puts the figures to anything between 0.2% and approximately 1% in the general population. The same applies for Bangladeshi immigrants to Europe, as studies have reported that the prevalence of HCV among Bangladeshi immigrants in Spain and UK is 0.09 and 0.6% respectively. The figures, as expected, are much higher among high-risk population, being as high as 24.8% among intravenous drug abusers. However, it is generally accepted that the prevalence HCV in this country is 0.84%.^7,8^ As with HBV, principal risk factors for the transmission of HCV in Bangladesh have also been identified as treatment from quacks, shaving and haircut in barber shops, body piercing, as well as vaccination against small pox, cholera, dental procedure, intravenous infusion, etc.^7^

There is a male predominance with males accounting for more than 70% HCV infections. More than 60% HCV-infected people in Bangladesh are between 30 and 50 years of age, and HCV is the second leading cause of CLD in Bangladesh, next only to HBV, accounting for 30% cases of liver cirrhosis and 17% cases of HCC. However, in recent times, we are experiencing a rise in fatty liver disease, which may bypass HCV in the next decade as the second leading cause of CLD.

The predominant GT of HCV in Bangladesh is GT 3 (50–89%), followed by GT 1 (8–29%).^9^ Until 2015, the mainstay of treatment of HCV in Bangladesh was double therapy consisting of Peg IFN and ribavirin (RIBA), with a reported SVR of 80% in GT 3. However, things have changed dramatically since February 2015, with the introduction of local, generic sofosbuvir (SOF) in our markets. We now also have local generic daclatasvir (DAC) and ladipasvir (LPV), in addition to generic Peg IFN and RIBA. This has opened a whole new spectrum of affordable treatment for HCV for Bangladeshi patients, which has been facilitated by a favorable government policy of withdrawal of tax and VAT on anti-HCV drugs. The cost of DAC in Bangladesh is less than USD 1.0, no wonder why we are seeing a sudden influx of health tourism in Bangladesh not only from the region but also from Europe and USA. Initial data from Bangladesh with triple therapy consisting of Peg IFN, SOF, and RIBA are showing promising results comparable with the published literature.^10^ Studies combining SOF plus DAC, Peg IFN, DAC plus RIBA, and SOF plus LPV are ongoing and data are expected to be released in 2016. However, the eradication of HCV from Bangladesh is still possibly a far cry, as it has been estimated that it will take more than 20 years to reduce the burden of HCV in India by 20%.

## HEPATITIS E VIRUS

Being a member of the developing world with significant poverty and poor hygienic conditions, not unexpectedly Bangladesh offers favorable conditions for nurturing hepatitis E virus (HEV) infection. Not only seasonal, but also sporadic, nonseasonal HEV outbreaks with significant mortality have been reported from Bangladesh.^11^ Not to mention that HEV remains the leading cause of acute hepatitis and also fatal conditions like acute hepatitis in pregnancy^2^ and acute chronic liver failure (ACLF) in this country.^12^ However, things are finally possibly moving in the positive direction, following the economic growth that Bangladesh has experienced in the recent years and the resultant improvement of socioeconomic, general living, and hygienic conditions. We are now seeing less cases of HEV during the monsoon in Bangladesh, and interestingly, unlike in 2013, in 2015 acute HBV infection bypassed acute HEV as the leading acute insult in ACLF in the country.^13^ It may therefore be predicted with confidence that HEV may become a bygone story in Bangladesh in the next couple of decades.

## References

[1] Mahtab MA, Rahman S, Foster G, Khan M, Karim MF, Solaiman S, Afroz S (2008). Epidemiology of hepatitis B VIRUS in Bangladeshi general population.. Hepatobiliary Pancreat Dis Int.

[2] Mahtab MA, Rahman S, Khan M, Karim MF (2009). HEV infection as an aetiologic factor for acute hepatitis: experience from a tertiary hospital in Bangladesh.. J Health Popul Nutr.

[3] Mahtab MA, Rahman S, Khan M, Kamal M, Karim MF, Ahmed F, Hussain MF, Podder PK (2007). Aetiology of chronic hepatitis in Bangladesh.. Indian J Gastroenterol.

[4] Afroz S, Mahtab MA, Rahman S, Khan M (2007). Hepatitis B virus is the leading cause of cirrhosis of liver in Bangladesh.. Hepato Int.

[5] Mahtab MA, Rahman S, Khan M, Ahmed F, Podder PK (2007). Aetiology of SOL of liver in Bangladesh.. Indian J Gastroenterol.

[6] Mahtab MA, Akbar SMF, Uddin H, Rahman S, Rubido JCA, Nieto GEG, Onji M, Mishiro S (2013). A phase III clinical trial with a nasal vaccine containing both HBsAg and HBcAg in patients with chronic hepatitis B.. J Hepatolo.

[7] Mahtab MA, Karim F, Foster G, Akbar SMF, Rahman S (2011). Prevalence and risk factors of asymptomatic HCV infection in Bangladesh.. J Clin Experiment Hepatol.

[8] Omata M, Kanda T, Yokosuka O, Crawford D, Mahtab MA, Wei L, Ibrahim A, Lau GKK, Sharma BC, Hamid SS, et al. (2015). Features of hepatitis C virus infection, current therapies and ongoing clinical trials in 10 Asian Pacific countries.. Hepatol Int.

[9] Mahtab MA, Rahman S, Khan M, Karim F, Sharif NM, Shrestha A (2008). Genotypes of HCV in Bangladesh: experience from a tertiary centre.. Hungarian Med J.

[10] Mahtab MA, Rahim MA, Alam SMN, Alam MA, Sarker JA, Khondaker MAF, Abedin MF, Moben AL, Foez SA, Uddin H, et al. (2015). RVR response in HCV related compensated CLD with triple therapy containing sofosbuvir in Bangladesh.. Hepatol Int.

[11] Rashid MH, Akbar SMF, Takahashi K, Mahtab MA, Khan MSI, Alim MA, Ekram ARMS, Khan MMR, Arai M, Mishiro S (2013). Epidemiological and molecular analyses of a non-seasonal outbreak of acute icteric hepatitis E in Bangladesh.. J Med Virol.

[12] Mahtab MA, Rahman S, Khan M, Karim MF (2009). Hepatitis E virus is the leading cause of acute-on-chronic liver disease: Experience from a tertiary centre in Bangladesh.. Hepato Biliar Pancreat Dis Int.

[13] Available from: www.aclf.in

